# Characteristics of Sleep Paralysis and Its Association With PTSD, Stress, and Other Lifestyle Variables Among the Population of Pakistan, a Cross‐Sectional Study

**DOI:** 10.1002/puh2.70292

**Published:** 2026-06-19

**Authors:** Alishba Javaid, Rabi Jan, Afifa Khan, Kanza Farhan, Thierry Nasibu Ntumba, Christian Tague, Aymar Akilimali

**Affiliations:** ^1^ Department of Medicine Islamic International Medical College Rawalpindi Pakistan; ^2^ Department of Medicine Jinnah Sindh Medical University Karachi Pakistan; ^3^ Faculty of Medicine Catholic University of Sapientia of Goma Goma, DR Congo; ^4^ General Reference Hospital La Charité Maternelle of Goma Goma, DR Congo; ^5^ Department of Research Medical Research Circle (MedReC) Bukavu, DR Congo

**Keywords:** caffeine intake, pakistan, perceived stress scale (PSS‐10), post‐traumatic stress disorder (PTSD) scale (PCL‐5), sleep paralysis (SP), sleep paralysis experiences and phenomenology questionnaire (SP‐EPQ), smoking

## Abstract

**Backgrounds:**

Sleep paralysis (SP) is a parasomnia disorder connected to being unable to move your body though the experiencing person remains conscious. Factors involving this disorder are stress, post‐traumatic stress disorder (PTSD), lifestyle factors including the amount of sleep, and the amount of caffeine consumed. Our study aim is to assess the symptoms, duration, time of occurrence, and body position during SP episodes and frequency of SP episodes with these variables.

**Methods:**

A total of 412 participants took part in this quantitative cross‐sectional study. Data were collected through online questionnaire. Ethical consideration was taken into account.

**Results:**

A significant correlation with a *p* value of less than 0.001 was discovered between the quantity of SP episodes and SP symptoms with inability to move the body has the highest frequency (44.7%) among people who experience SP (48.1%). Significant association was found between frequency of SP episodes with duration, that is, fewer seconds (20.9%), occurrence of SP upon falling asleep (23.8%), and body position during SP, that is, on back (26.5%) having *p* value of less than 0.001, respectively. A noteworthy correlation was discovered between the frequency of SP episodes, PTSD, and stress having a *p* value of 0.009. Significant association was also found between frequency of SP episodes with caffeine intake (38.1%) and smoking (2.9%) having a *p* value of <0.001.

**Conclusions:**

This study suggests a relationship between SP and lifestyle and psychological factors.

## Graphical abstract

Sleep paralysis often strikes when falling asleep in a supine position; its frequency links strongly with lifestyle factors. Both sleep timing and posture significantly influence episodes. Research involving 412 Pakistani participants highlights these correlations, emphasizing how daily habits impact this condition's occurrence.

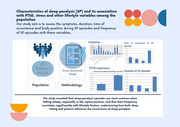

## 1 Introduction

According to the latest version of the International Classification of Sleep Disorders (ICSD‐3rd‐TR), sleep paralysis (SP), also referred to as hypnagogic SP, is defined as a transient inability to move the body due to muscle atonia occurring either at sleep onset or upon awakening, whereas the individual remains conscious [1, 2].

Episodes typically last from a few seconds to several minutes [3, 4] and occur during rapid eye movement (REM) sleep. REM sleep is a distinct physiological stage characterized by vivid, often recallable dreams, REMs, and heightened cortical activity, accompanied by near‐complete skeletal muscle atonia that serves as a protective mechanism to prevent individuals from physically acting out dreams ([5, 6]; https://doi.org/10.1111/jsr.14342). During this stage, complete muscle atonia occurs—except for ocular and respiratory muscles—preventing individuals from physically acting out their dreams, a protective mechanism against potential injury when the motor cortex is highly active [7]. SP is considered a pathological manifestation of disrupted REM sleep regulation, wherein elements of REM atonia persist into wakefulness while consciousness is preserved.

SP episodes are frequently accompanied by vivid and often distressing hallucinations. Approximately 75% of affected individuals report such experiences alongside muscle atonia. These hallucinations are commonly categorized into three types: intruder hallucinations, incubus hallucinations, and visual‐motor (V‐M) hallucinations. Intruder hallucinations involve sensations of a threatening presence in the room, often accompanied by fear, auditory disturbances, and visual perceptions such as dark figures or shadows. Incubus hallucinations are marked by sensations of chest pressure, feelings of choking or suffocation, and in some cases, visual images of a figure sitting on the chest. V‐M hallucinations are characterized by out‐of‐body experiences (OBE), autoscopy (seeing oneself from an external perspective), and sensations of floating or spinning [1, 7]. Among these, intruder and incubus hallucinations are the most prevalent, with 80%–90% of individuals experiencing intense fear during episodes [5]. Conversely, V‐M hallucinations may occasionally be associated with pleasant emotions, and studies indicate that approximately 16%–17% of individuals report positive SP episodes [5, 8].

Epidemiological studies estimate the global lifetime prevalence of SP at 7.6% (2). Although prevalence does not significantly vary by age [9, 10], it is notably higher among student populations, affecting approximately 28% of young adults, and even more pronounced among psychiatric patients, with rates approaching 32% [3]. Evidence suggests that individuals who experience SP earlier in life tend to have more frequent episodes [3, 11]. Although the precise mechanisms underlying its occurrence remain unclear, several psychosocial and behavioral factors have been implicated. These include stress, anxiety, post‐traumatic stress disorder (PTSD), and irregular sleep patterns [9]. Lifestyle factors such as excessive caffeine intake, substance use, and poor sleep hygiene also contribute [12]. SP is particularly prevalent among individuals with preexisting psychiatric conditions, with studies reporting that 27.8%–76% of individuals with PTSD have experienced at least one SP episode, making PTSD the most common psychiatric comorbidity [1, 9].

SP may occur in isolation, termed isolated sleep paralysis (ISP), or in association with medical conditions such as narcolepsy and seizure disorders [1, 13]. Recurrent or repeated isolated sleep paralysis (RISP) refers to individuals experiencing multiple ISP episodes, often linked to sleep–wake regulation disturbances [1, 14].

Given the distressing and often bizarre nature of hallucinations associated with SP, the phenomenon has been historically explained through supernatural or cultural interpretations (for instance, in Egypt, episodes are attributed to “jinn”; in Cambodia, to the belief of “a ghost pressing down on the chest”; and in Mexico, to the phrase “the dead climbed on top of me”), heightening the distress related to such episodes [15, 16, 17].

Overall, the prevalence and experience of SP appear to be influenced by a combination of social, psychological, cultural, and health‐related factors [1, 2, 18]. The present research aims to address gaps in understanding the phenomenology of SP and to explore its associations with lifestyle factors, stress, and PTSD within Pakistan's general population.

## 2 Materials and Methodology

The quantitative cross‐sectional study was carried out from December 2023 to February 2024 among those Pakistani citizens who can understand English reading and writing. On the basis of random probability sampling, 412 participants took part in this study. Using the “Rao soft” sample size calculator, a sample size of 385 was determined. Participants whose ages were 18 and above were included in this study while people below age 18 and who are illiterate were excluded. The link to the survey was shared by messaging apps and social media sites and participation was entirely voluntary. SP was defined according to ICSD‐3‐TR, participants were instructed to label it only when there is muscle atonia along with conscious level which differentiates it from REM sleep behavior disorder, nightmares, and insomnia. Data were collected via online Google forms by using a 95% confidence interval with 5% margin of error. Ethical consideration was taken into account. It was approved by IRC “Ref No. Riphah/IIMC/IRC/23/3118.” Consent forms were filled by participants before taking part in research project. For data collection, a detailed questionnaire consisting of open‐ and closed‐ended questions related to demographic aspects, the PTSD Checklist (PCL‐5), the Perceived Stress Scale (PSS‐10), and the Sleep Paralysis Experiences and Phenomenology Questionnaire (SP‐EPQ) was completed by all study participants. Demographic section included the personal data and lifestyle variables, that is, sleep, caffeine intake, and smoking related questions. Health data include presence of any psychiatric condition and medication taken. Next section was SP‐EPQ which included questions related to frequency of SP episodes, characteristics of symptoms, episodes duration and occurrence time, and body position during SP episodes. It started with the question “have you ever experienced SP in your lifetime.” Upon a positive response, participants were then asked to explain the experience thus to validate it that the experience was in fact SP. Cronbach's alpha was used for the validation of these tools in Pakistani population. The PTSD checklist for DSM‐5 (PCL‐5) was used in this study where 20 items on the checklist were presented to rate the intensity of symptoms on a five‐point Likert scale that goes from 0 (not at all) to 4 (very). There are strong psychometric qualities to this scale. The research's Cronbach's alpha coefficient was 0.969. Furthermore, the PSS was evaluated using the PSS‐10 scale which consists of ten questions covering subjective feelings regarding various issues, stress‐related occurrences, and coping mechanisms. This scale has strong psychometric properties. The Cronbach's alpha coefficient for this scale was 0.91. Questionnaire with incomplete information and those who do not met the criteria of inclusion and exclusion were excluded from this study.

### 2.1 Statistical Analysis

To ascertain a relation between the categorical variables, that is, SP, stress, PTSD, and lifestyle variables, the nonparametric Chi‐square test was utilized. Multivariate logistic regression was applied to adjust for potential confounders including age, gender, stress levels, caffeine use, and smoking status. For analysis, IBM SPSS 21 was used. Excel was utilized to create graphs.

## 3 Results

Our study focused on SP's characteristics and how it relates to stress, PTSD, and other lifestyle factors in Pakistan. ***p* values less than 0.05 were regarded as statistically significant**.

**FIGURE 1:**
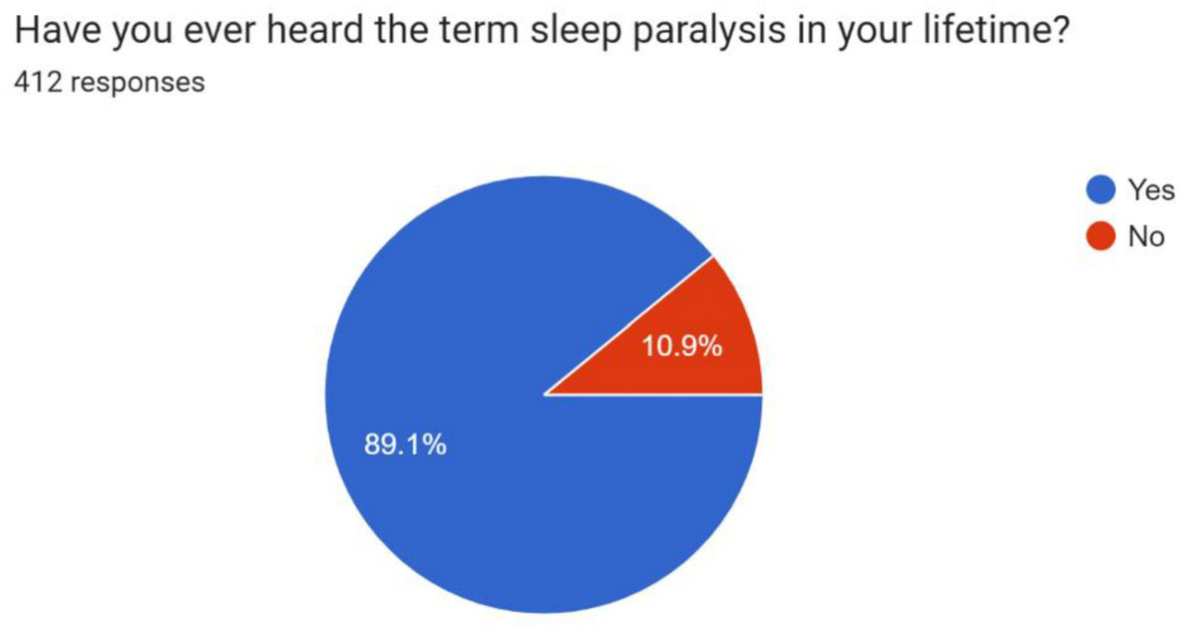
Awareness's regarding sleep paralysis.

**FIGURE 2:**
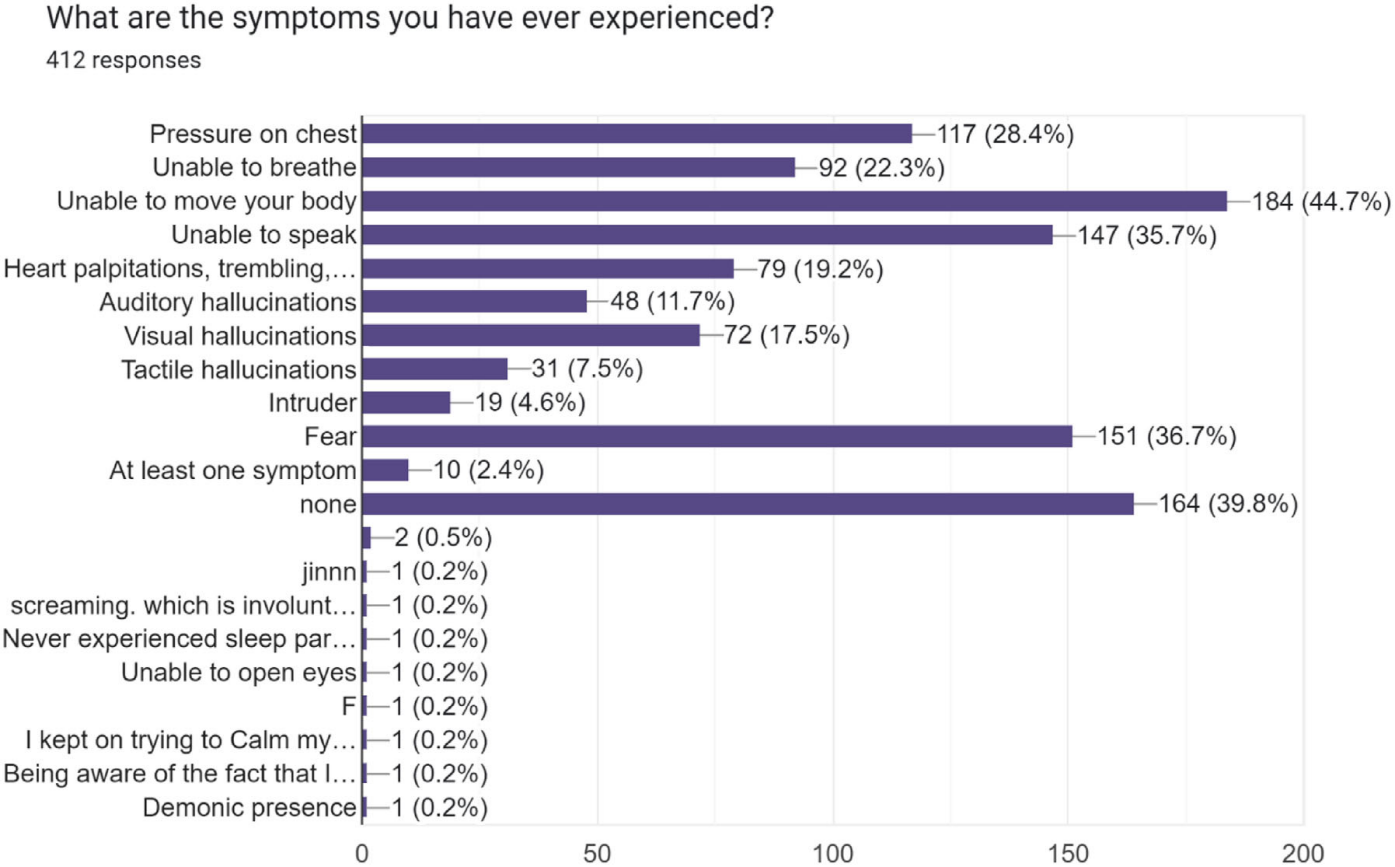
Symptoms.

**FIGURE 3:**
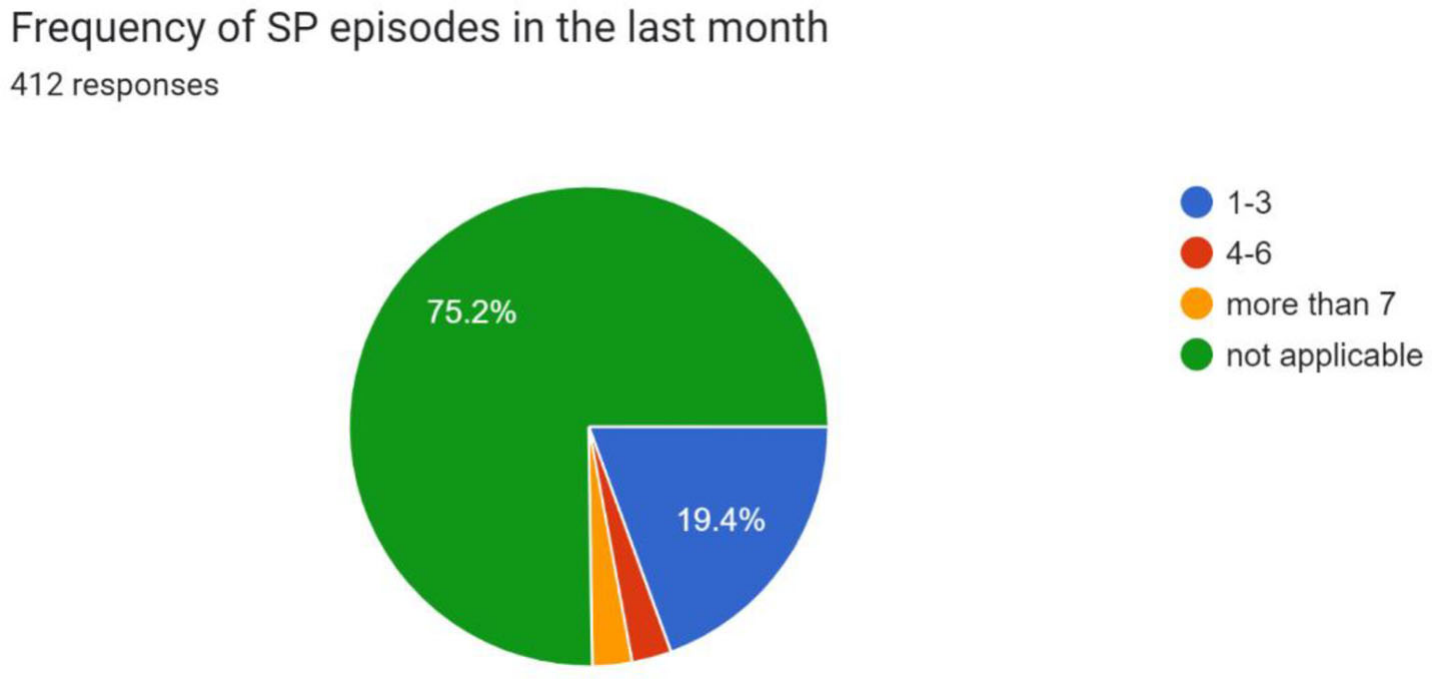
Frequency of SP episodes in last month. SP, sleep paralysis.

**FIGURE 4:**
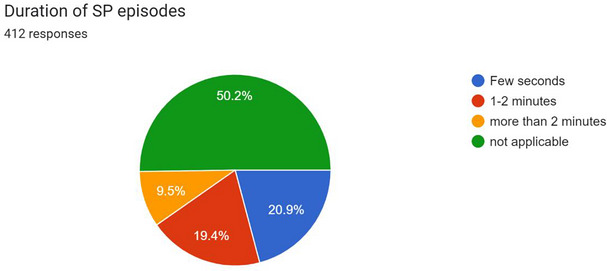
Duration of SP episodes. SP, sleep paralysis.

As shown in Figure 1, out of *N* = 412 respondents, 198 (48.1%) have experienced SP and 214 (51.9%) have not experienced SP in their lifetime. As shown in Figure 2 symptoms experienced by respondents mostly are unable to move your body 184 (44.7%) followed by fear 151 (36.7%), unable to speak 147 (35.7%), pressure on chest 117 (28.4%), unable to breathe 92 (22.3%), heart palpitations, trembling, numbness, sweating and chills 79 (19.2%), visual hallucinations 72 (17.5%), auditory hallucinations 48 (11.7%), tactile hallucinations 31 (7.5%), intruder 19 (4.6%), at least one symptom 10 (2.4%) other constitutes 2.1%. As shown in Figure 3, frequency of 1–3 SP episodes in the last month is 19.4%, 4–6 episodes are 2.7%, and more than 7 episodes are 2.7%. Duration of few seconds 20.9% is higher in number followed by 1–2 min 19.4% and more than 2 min which is 9.5%, Figure 4.

**FIGURE 5:**
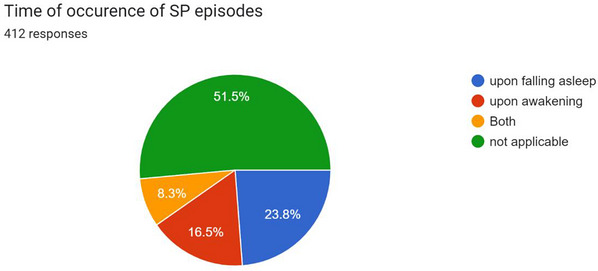
Time of occurrence of SP episodes. SP, sleep paralysis.

**FIGURE 6:**
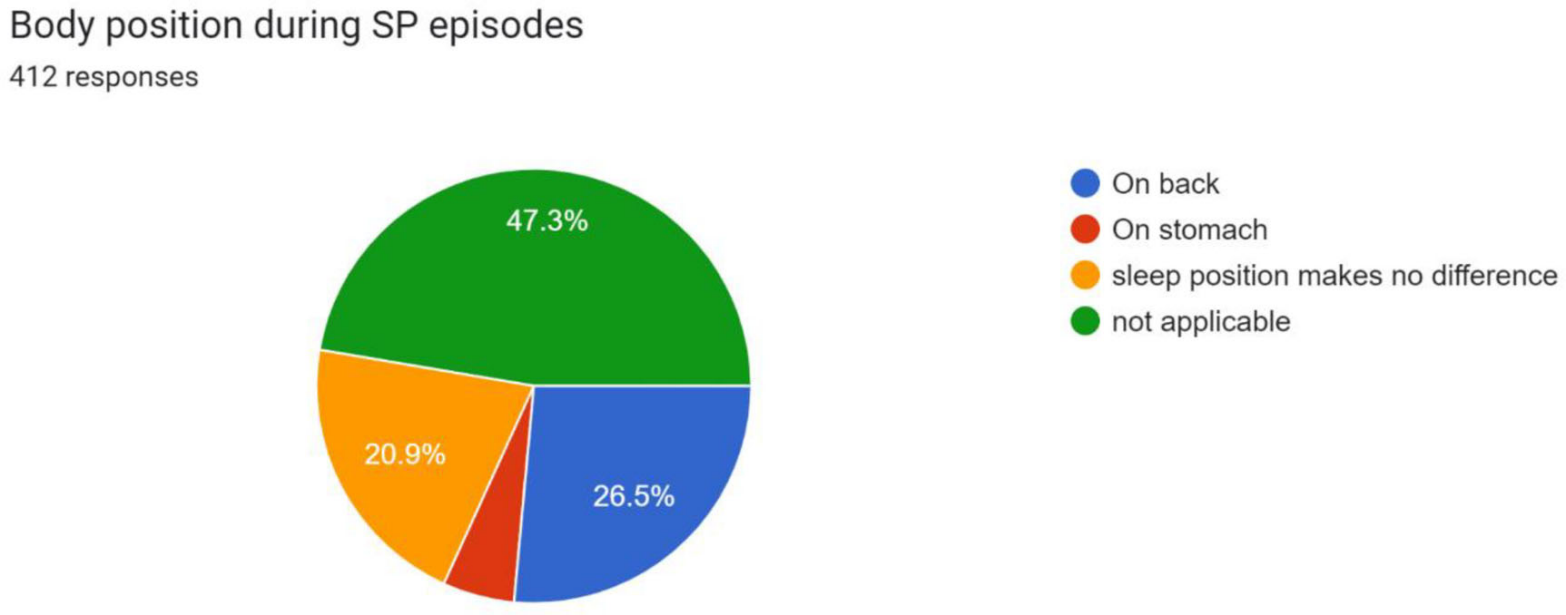
Body position during SP episode. SP, sleep paralysis.

**FIGURE 7:**
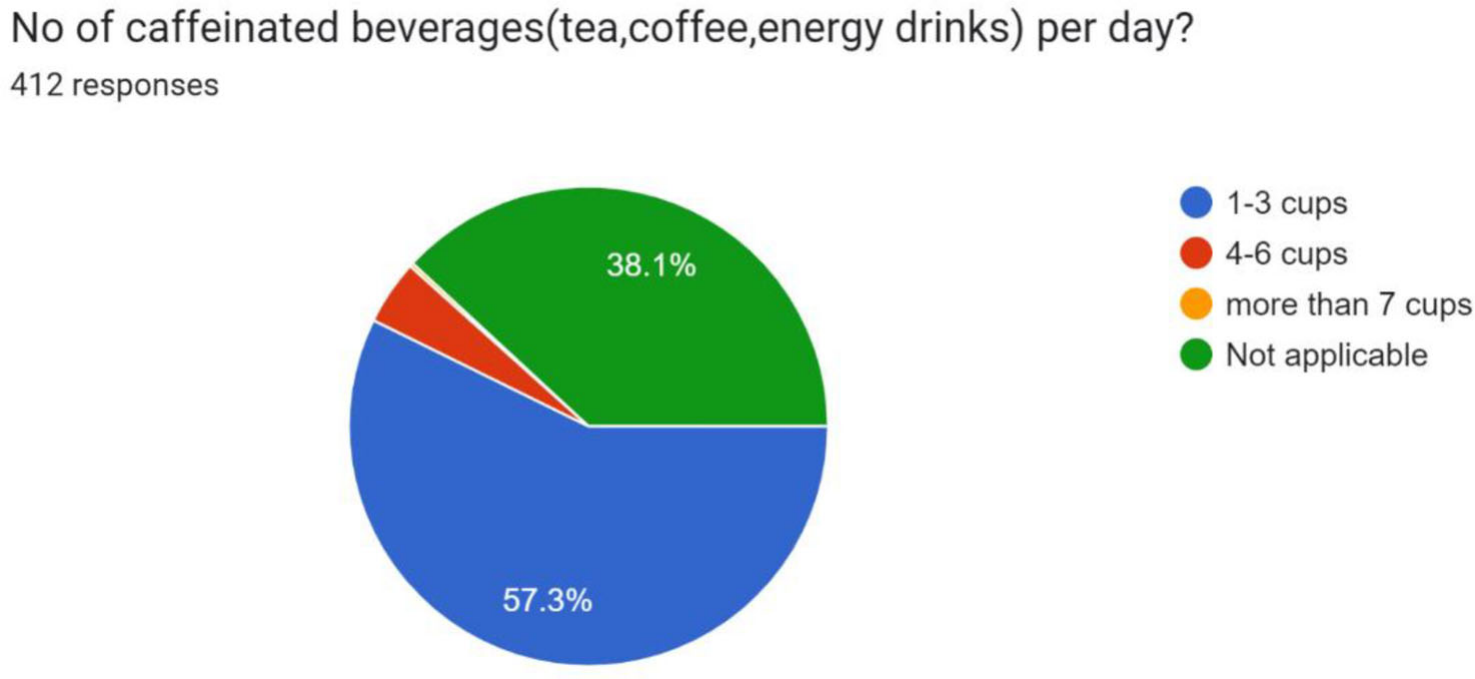
Beverages’ quantity per day.

**FIGURE 8:**
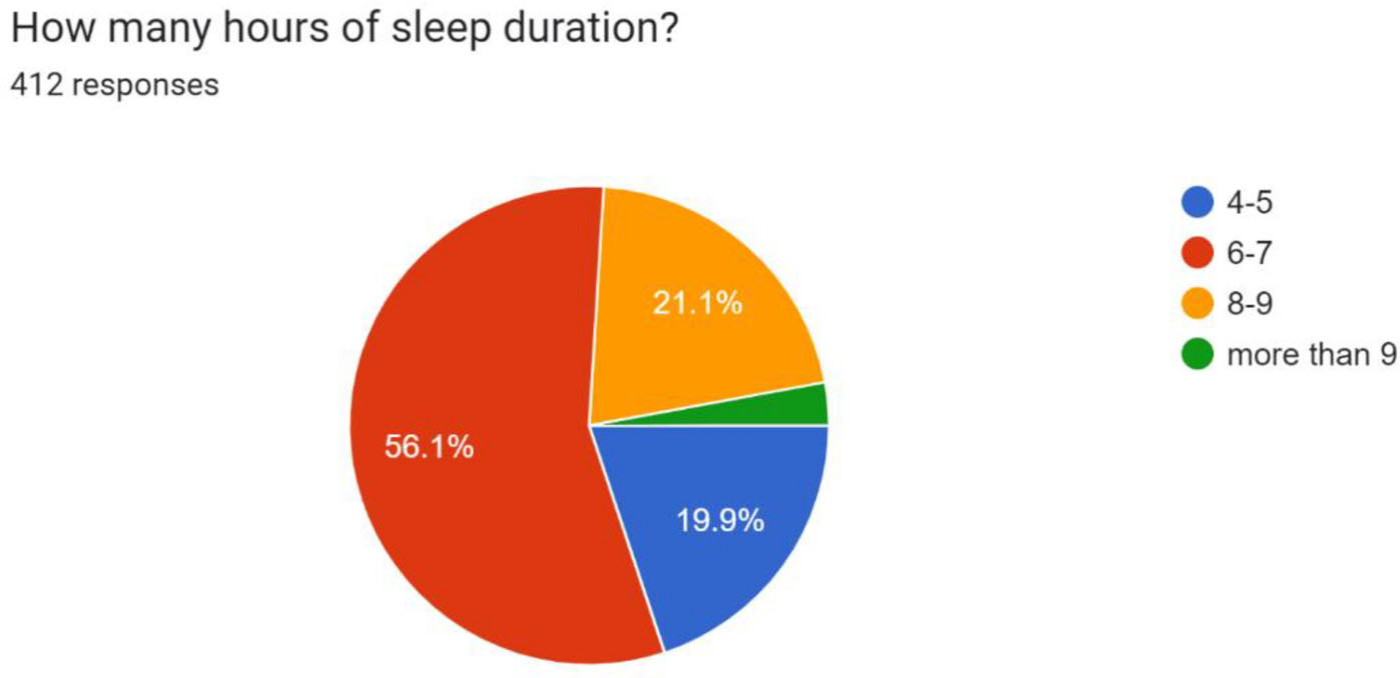
Hours of sleep duration.

**FIGURE 9:**
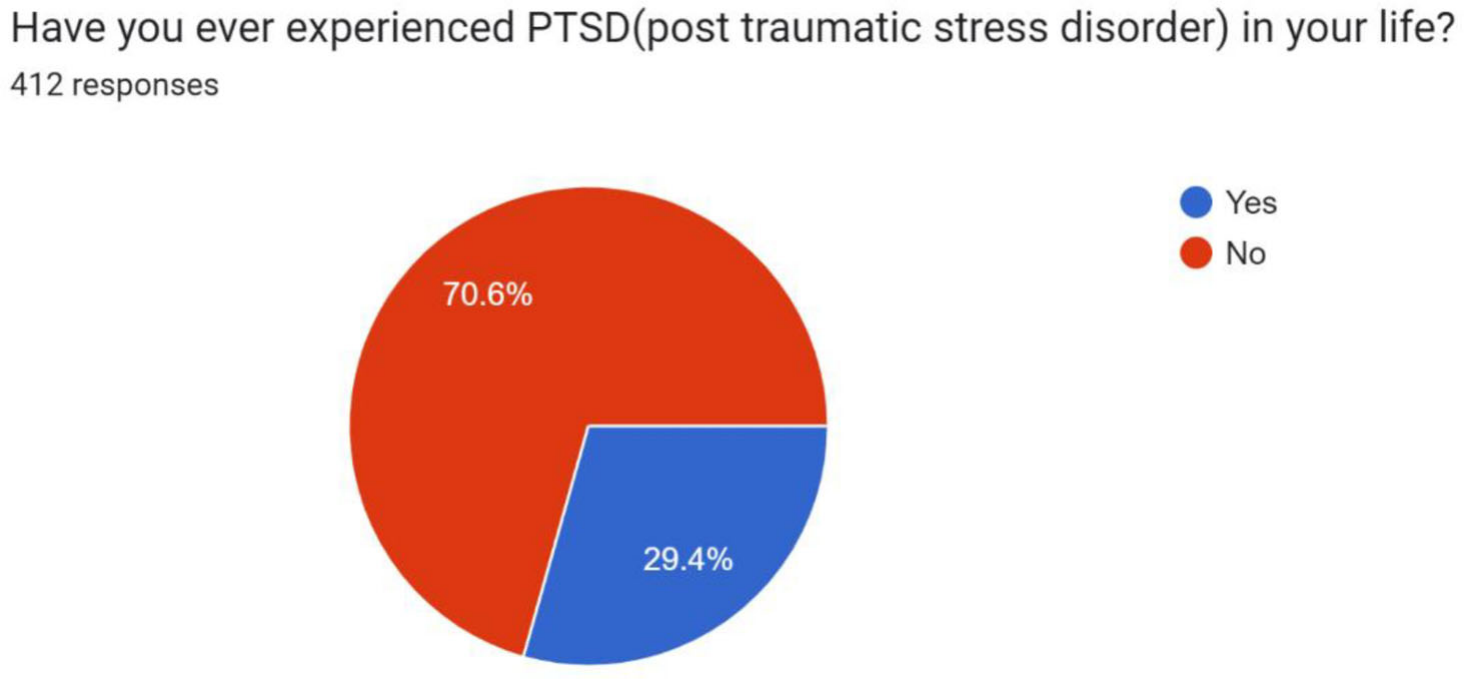
PTSD experience.

The percentage of SP episodes that occur right before bedtime is most which is 23.8%, followed by upon awakening 16.5% and then upon both which is 8.3%, Figure 5. People experience SP mostly when they lie on back 26.5% followed by sleep position makes no difference 20.9% and then on stomach 5.3%, Figure 6. People who consume Figure 7, caffeinated beverages 1–3 cups are higher in number which is 38.1% followed by 4–6 cups 4.4%. People who smoke less than 1 pack per day are higher in number 2.9% followed by 1 pack and less than 1 pack which are 1.5% and 0.7%, respectively. Overall, 94.9% are nonsmokers in our sample size population. In our study as Figure 8 shows that 56.1% had 6–7 h of sleep, 19.9% had 4–5 h of sleep, 22.1% had 8–9 h of sleep, and 2.9% had more than 9 h of sleep. Overall, 19.2% encounter these SP symptoms when they go to bed later than their usual time. Overall, 29.4% had experienced PTSD in their lifetime shown in Figure 9. Overall, 62.6% of the sample population thinks that stress increases the frequency of SP.

A strong correlation was discovered between frequency of SP and symptoms of SP episodes, duration of SP episodes, time of occurrence of SP episodes, and body position during SP episodes **(*p* value <0.001 for each analysis)**. There was a significant correlation discovered between frequency of SP episodes with overall mean PTSD **(*p* value = 0.009)**. Significant correlation was also found between frequency of SP episodes with stress **(*p* value = 0.009)**.

A significant connection was found between the frequency of SP episodes and staying up later than normal **(*p* value <0.001)**. Despite this, a significant association was observed between the frequency of SP episodes and the amount of sleep **(*p* value = 0.014). A statistically significant relationship was observed between the frequency of SP episodes and both smoking and caffeine intake (*p* value <0.001 for both)**.

Multivariate logistic regression was applied to adjust for potential confounders including age, gender, stress levels, caffeine use, and smoking status.

A strong and statistically significant association was observed between caffeine intake and the occurrence of SP episodes. Each increase in caffeine consumption category (e.g., none to 1–3 cups, 1–3 to 4–6 cups) was associated with more than a two‐fold increase in odds of SP (OR = 2.14, 95% CI: 1.42–3.21, *p* < 0.001). A sensitivity analysis treating caffeine use as a binary variable (yes vs. no) yielded consistent results (OR = 2.36, 95% CI: 1.51–3.69, *p* < 0.001).

Stress levels were also significantly associated with SP. For each one‐point increase on the stress scale (0–4), the odds of reporting SP increased by approximately 40% (OR = 1.40, 95% CI: 1.11–1.77, *p* = 0.004).

Gender showed a borderline association (OR = 0.57, 95% CI: 0.31–1.07, *p* = 0.082), suggesting possible differences between categories, though this did not reach statistical significance.

By contrast, age (OR = 1.00, 95% CI: 0.97–1.04, *p* = 0.864) and smoking (OR = 0.62, 95% CI: 0.29–1.33, *p* = 0.218) were not significantly associated with SP episodes.

## 4 Discussion

This study investigated the prevalence and characteristics of SP and its associations with stress, PTSD, and lifestyle variables in a sample of the Pakistani population. Nearly half of the respondents (48.1%) reported experiencing SP, a prevalence that is considerably higher than the global estimate of 7.6% reported in systematic reviews. **This elevated prevalence may also reflect participation bias, as individuals who have experienced SP—particularly recurrent or distressing episodes—may be more likely to engage in such studies**. Several factors may account for this elevated prevalence, including methodological issues such as reliance on self‐reported online surveys, cultural differences in how SP is interpreted, and the high levels of stress and trauma exposure in the region. Similar findings of elevated prevalence in high‐stress populations, such as students and psychiatric patients, have been **documented, with evidence suggesting a high stress burden among student populations**.

Consistent with earlier research, the most commonly reported symptoms in this study were inability to move, intense fear, speech arrest, and chest pressure [1]. These symptoms reflect the typical REM‐related atonia and distressing hallucinations described in the literature. Although hallucinations such as intruder and incubus experiences were less frequently endorsed in our sample, they are known to contribute to heightened distress in SP episodes. Importantly, the severity of reported symptoms was positively correlated with the frequency of episodes, supporting the notion that recurrent SP is not only more disruptive but also more symptomatic. This aligns with prior findings among both medical students in Pakistan and student populations in Europe.

A key finding of our study is the association between SP frequency and sleeping position. Over one‐quarter of participants reported that episodes occurred more often while lying supine. Previous research suggests that this posture may exacerbate airway obstruction and contribute to a sense of pressure on the chest, thereby increasing the likelihood of SP [19]. Cross‐cultural comparisons have similarly highlighted the role of supine position in triggering SP, regardless of participants’ habitual sleeping posture [20]. These findings reinforce the recommendation that individuals prone to SP may benefit from positional sleep interventions.

The duration and timing of SP episodes also showed significant associations with frequency. Episodes were more commonly reported upon sleep onset than awakening, a pattern consistent with prior studies that have linked sleep‐onset REM periods with greater SP susceptibility. Episodes lasting longer than 2 min, though less frequent, were perceived as particularly distressing, underscoring the importance of early recognition and management [21].

**Unhealthy lifestyle factors** emerged as significant correlates of SP. High caffeine consumption and cigarette smoking were both positively associated with SP frequency, consistent with evidence that stimulants and nicotine disrupt normal REM cycles and increase sleep fragmentation [12]. These findings suggest that behavioral modifications, such as reducing stimulant intake, may serve as practical preventive strategies. Additionally, a study conducted on student in Poland showed that there is correlation between these two factors [22].

Psychological variables, particularly stress and PTSD, were strongly associated with SP in our study. Nearly one‐third of respondents reported PTSD symptoms, and both PTSD and perceived stress scores correlated with higher SP frequency. This is consistent with prior findings among traumatized populations, including Cambodian refugees and university students, where PTSD has been identified as a major risk factor for SP [15]. Importantly, although our cross‐sectional design cannot establish causality, the observed associations underscore the need for clinicians to consider comorbid stress and trauma symptoms in patients reporting SP [14, 19].

Cultural beliefs may further shape the perception and reporting of SP in Pakistan. As in many societies, SP has historically been explained through supernatural interpretations, such as the presence of jinn or other entities. Such interpretations can heighten fear and distress during episodes, influencing both prevalence estimates and the severity of subjective experience. This cultural lens should be considered in public health interventions, where psychoeducation may reduce stigma and promote healthier coping strategies.

## 5 Limitations

This study is subject to several limitations. First, there is a chance of recall and social desirability biases when self‐reported data are used. There is still a chance of misclassification because of the intricacy of the phenomena and the dependence on subjective reporting, even though participants were told to differentiate SP from other sleep disturbances in accordance with the ICSD‐3‐TR definition. Moreover, this study's definition of SP was based on participant self‐recognition rather than clinical evaluation, which would make it less comparable to research using standardized diagnostic techniques. Last but not least, the adoption of a non‐probability, online sampling technique limits generalizability because participation was contingent on digital literacy, internet connection, and voluntary engagement, which lowers representativeness.

Additionally, the high prevalence of SP observed in this study may be influenced by participation bias, as individuals who have experienced SP—particularly recurrent or distressing episodes—may have been more likely to participate. Furthermore, the overrepresentation of student populations, who are known to experience higher stress levels [23], may have further contributed to the elevated prevalence estimates.

## 6 Recommendations

Lifestyle modifications must be taken into consideration.

People should avoid stimulants like caffeinated beverages and smoking.

It is recommended for people to sleep on the side position instead of lying on their back. Psychiatric conditions must be taken into account and people should properly get themselves checked by psychiatrists.

People should limit stresses in their life by getting involved in activities outside their daily routine, by practicing yoga and meditation exercises.

## 7 Conclusions

Frequency of SP shows significant association with symptoms of SP, duration, time of occurrence, and body position during SP episodes among the population of Pakistan. Frequency of SP episodes shows significant findings with caffeine consumption and smoking. Frequency of SP episodes shows significant association with PSS and PTSD.

## Author Contributions

**Alishba Javaid**: conceptualization, methodology, writing – review and editing, writing – original draft. **Rabi Jan**: validation, visualization, writing – original draft. **Afifa Khan**: writing – review and editing, investigation. **Kanza Farhan**: funding acquisition, writing – review and editing. **Thierry Nasibu Ntumba**: writing – review and editing, resources. **Christian Tague**: validation, project administration, writing – review and editing, writing – original draft. **Aymar Akilimali**: project administration, writing – original draft.

## Funding

The authors have nothing to report.

## Conflicts of Interest

The authors declare no conflicts of interest.

## Data Availability

The data that support the findings of this study are available in the Supporting Information of this article.
